# Understanding user perceptions of DeepSeek: insights from sentiment, topic and network analysis using a Reddit-based study

**DOI:** 10.3389/frai.2025.1703949

**Published:** 2026-01-06

**Authors:** Naisarg Patel, Rajesh Sharma, Prakash Lingasamy, Vino Sundararajan, Sajitha Lulu Sudhakaran, Vijayachitra Modhukur

**Affiliations:** 1 Integrative Multiomics Lab, School of Bio Sciences and Technology, Vellore Institute of Technology, Vellore, Tamil Nadu, India; 2 School of AI and Computer Science, Plaksha University, Sahibzada Ajit Singh Nagar, Punjab, India; 3 Institute of Computer Science, University of Tartu, Tartu, Estonia; 4 Department of Obstetrics and Gynecology, Institute of Clinical Medicine, University of Tartu, Tartu, Estonia; 5 Nalam Biosciences OÜ, Tartu, Estonia

**Keywords:** Deepseek, generative AI, Reddit, natural language processing, sentiment analysis, topic modeling, network analysis

## Abstract

**Introduction:**

The launch of DeepSeek, a Chinese open-source generative AI model, generated substantial discussion regarding its capabilities and implications. The r/deepseek subreddit emerged as a key forum for real-time public evaluation. Analyzing this discourse is essential for understanding the sociotechnical perceptions shaping the integration of emerging AI systems.

**Methods:**

We analyzed 46,649 posts and comments from r/deepseek (January–May 2025) using a computational framework combining VADER sentiment analysis, Hartmann emotion classification, BERTopic for thematic modeling, hyperlink extraction, and directed network analysis. Data preprocessing included cleaning, normalization, and lemmatization. We also examined correlations between sentiment/emotion scores and dominant topics.

**Results:**

Sentiment was predominantly positive (posts: 47.23%; comments: 44.26%), with neutral sentiment comprising ~30% of content. The most frequent emotion was neutrality, followed by surprise and fear, indicating ambivalent user reactions. Prominent topics included open-source AI models, DeepSeek usage, device compatibility, comparisons with ChatGPT, and censorship concerns. Hyperlink analysis indicated strong engagement with GitHub, Hugging Face, and DeepSeek’s own services. Network analysis revealed a fragmented but active community, depicting Open-Source AI Models as the most cohesive cluster.

**Discussion:**

Community discourse framed DeepSeek as both a technical tool and a geopolitical issue. Enthusiasm centered on its performance, accessibility, and open-source nature, while concerns were voiced about censorship, data privacy, and potential ideological influence. The integrated analysis shows that collective perception emerged through decentralized, dialogic engagement, reflecting broader sociotechnical tensions related to openness, trust, and legitimacy in global AI development.

## 1 Introduction

The proliferation of online communities has transformed how individuals share opinions, exchange ideas, and provide feedback on emerging technologies. Platforms like Reddit, hosting millions of active users, generate diverse, unstructured content that serves as a valuable resource for understanding public sentiment and preferences (Talafidaryani and Moro, 2024). For technology developers, these platforms offer a wealth of data to inform product enhancements, improve user satisfaction, and address concerns. Natural Language Processing (NLP) techniques, such as sentiment analysis and topic modeling, enable efficient analysis of this data, converting raw text into actionable insights (Pang and Lee, 2008).

Sentiment analysis classifies text into positive, negative, or neutral categories based on emotional tone, proving effective for social media and customer feedback studies (Xu et al., 2022). Tools like VADER excel at handling informal language prevalent on Reddit. Topic modeling identifies recurring themes in text corpora, with methods like BERTopic uncovering discussion topics (Silva et al., 2024). Combining these approaches provides a dual perspective by quantifying user emotions and identifying discussion themes, thus offering a comprehensive view of community dynamics. In most previous studies, analytical dimensions, notably emotional (sentiment), thematic (topics), and relational (interaction) have been treated as distinct and separate strands (Albalawi et al., 2020). While only a handful of studies have sought to integrate them into a unified computational framework to examine how affect, meaning, and social connection intertwine in online discussions about artificial intelligence (AI) (Rueger et al., 2023; Molenaar et al., 2023).

DeepSeek, an AI-powered platform for advanced search and data analysis, has gained attention for its innovative features (Poo, 2025). Developed in China and released as an open-source language model, DeepSeek’s origin has sparked global discussion about transparency and trust in AI ecosystems (Huynh and Aichner, 2025). Its Chinese origin positions it at the intersection of innovation and geopolitical perception (White et al., 2024), raising questions about how “open source” is interpreted across regulatory and cultural contexts. The subreddit dedicated to it (r/deepseek), is a hub for enthusiasts and professionals and a prime space for sharing experiences and critiques. Reddit’s features of anonymity, upvoting/downvoting mechanisms, and asynchronous discussion threads make it ideal for exploring collective sense-making around emerging technologies (Muljana et al., 2022; Huang et al., 2025). These features encourage open discussions that contrast with more curated, identity-linked conversations on X (Oldemburgo et al., 2024; Utz and Breuer, 2019). Analyzing this community’s content can reveal how DeepSeek is perceived, what users value, and where improvements are needed. Given the volume and unstructured nature of Reddit data, manual analysis is infeasible, necessitating automated NLP methods.

While sentiment analysis and topic modeling have been applied to platforms like Twitter (Wang et al., 2022) and broader subreddits (Li et al., 2023), their integration with network and hyperlink analyses to capture emotional, thematic and relational structures remains uncommon, particularly in technology-specific communities like DeepSeek’s subreddit remains underexplored. Few studies integrate sentiment and topic analyses to examine how emotional tones correlate with discussion themes in such contexts, limiting insights into user perceptions of newer platforms like DeepSeek (Rustam et al., 2021). This study addresses this gap by applying VADER for sentiment analysis and BERTopic for topic modeling to the DeepSeek subreddit, aiming to: (1) assess community sentiment; (2) identify key discussion topics; (3) explore sentiment-topic relationships; and (4) provide recommendations for platform improvement.

## 2 Related works

The rapid growth of online communities like Reddit provide researchers with a dearth of datasets to explore user interactions and discussions around emerging technologies. This study builds on prior work in social media analysis, sentiment analysis, topic modeling, and network analysis, while addressing a gap in studying AI-focused communities like r/deepseek. By integrating multiple computational methods, this work aims to explore affective (sentiment and emotion), thematic (topics), and relational (interaction) dimensions, thereby providing a holistic perspective on how users engage with and interpret emerging AI technologies (Rueger et al., 2023). While sentiment, topic, and network analyses are well established individually, few studies have combined them to examine online communities (Albalawi et al., 2020). Integrating these perspectives allows exploration of emotional tone, thematic focus, and interaction structure, capturing how collective meaning-making unfolds in digital spaces.

### 2.1 Sentiment analysis in social media

Sentiment analysis has been widely used to capture user attitudes in online environments. A foundational overview was provided by Pang and Lee (2008), outlined core techniques for large-scale opinion classification (Pang and Lee, 2008). Building on this foundation, the Valence Aware Dictionary and Sentiment Reasoner (VADER) became a widely adopted tool due to its ability to process informal language, emojis, and slang that characterize social media text (Hutto and Gilbert, 2014).

Applications of sentiment analysis demonstrate its value in capturing public perceptions across diverse domains. For example, several studies analyzed Twitter data to examine attitudes toward technology adoption (Al-Daihani, 2016; Wamba and Carter, 2013), while Abouei et al. (2025) studied Reddit’s emotional expressions to reveal collective emotional dynamics. Despite the advancement, challenges remain in handling sarcasm, heterogeneous text, and context shifts (Yadav and Vishwakarma, 2019; Zimbra et al., 2018). Notably, research on sentiment within AI communities, such as r/deepseek, remains underexplored, raising questions about how users articulate their emotional responses to the technical and ethical dimensions of emerging AI systems.

### 2.2 Topic modeling for thematic insights

Topic modeling has become an established approach for providing insights on latent themes in text data collections. Latent Dirichlet Allocation (LDA), remains a widely used method for identifying topic distributions in documents (Blei et al., 2025). However, LDA produces fragmented topics when applied to short, noisy texts like social media. To address these limitations, BERTopic combines transformer-based embeddings with class-based TF–IDF to generate more coherent and interpretable topics (Grootendorst, 2022). Applications of topic modeling in social media research demonstrate its potential for thematic analysis. Cai et al. (2023) applied topic modeling to Reddit discussions of ChatGPT, uncovering themes of productivity, creativity, and ethical concerns, linking these with public sentiment (Liu et al., 2023). Similarly, Wang et al. (2021) used LDA and BERTopic on COVID-19 Twitter discussions to reveal evolving themes in public health discourse (Wang et al., 2021). Despite these advancements, there remains a scarcity of topic-based exploration in AI-focused subreddits, particularly where discussions are both technically detailed and socially reflective.

### 2.3 Network analysis of online interactions

Prior research has applied network analysis to Reddit in multiple ways, highlighting its potential for understanding online discourse and interaction. For instance, a study on a voting-focused subreddit examined community dynamics using network structures to capture patterns of participation and influence (Goglia and Vega, 2024). Other work constructed networks between subreddits based on crossposting behavior, demonstrating how content flows across different communities (Sawicki et al., 2023). Reddit networks have also been leveraged in agent-based simulations to model interactions between heterogeneous users, moderators, and subreddits (Murdock et al., 2023). At the discussion level, hierarchical comment thread structures have been analyzed to show how top-level comments generate subtopics (Weninger et al., 2013), and role-based studies revealed the recurring presence of the “answer-person” within Reddit communities (Buntain and Golbeck, 2014). Some approaches constructed undirected networks to explore relationships among users without specifying interaction directionality (Shrestha et al., 2020). Building on these works, our study extends network-based approaches by incorporating sentiment analysis and topic modeling simultaneously. Specifically, we create a directed network that captures both posting and commenting behavior, enabling us to identify not only who contributes discussion topics but also how other users engage with them, while considering the overall sentiment expressed across the community.

### 2.4 Hyperlink analysis in online communities

Hyperlinks on Reddit have been studied as an important mechanism for understanding information flows and inter-community dynamics. Kumar et al. (2018) examined hyperlinks to identify inter-subreddit relations, showing how shared links connect otherwise separate communities. Rohde et al. (2021) found that 4–5% of posts contained hyperlinks, though they did not pursue further analysis of the linked content. Building on this, Krohn and Weninger (2022) focused exclusively on inter-subreddit link analysis, mapping how hyperlinks serve as bridges between communities. Other studies have shifted attention to the role of hyperlinking in user behavior, for instance Cauteruccio et al. (2020) classified Reddit authors into types such as “knowledge brokers,” who primarily share links to external resources, while Özkula et al. (2022) highlighted the central role of hyperlinks in online debates and cross-platform exchanges. Building on these previous studies, our work analyzes all hyperlinks shared within a single subreddit r/deepseek and identifies the role of the external sources and platforms users rely on most. This approach highlights the ecosystem of external websites and tools that is discussed and used by the AI community.

### 2.5 Studies on AI and technology communities

Research on AI technologies in online communities has been largely focusing on ChatGPT. For example, Xu et al. (2024) examined Reddit conversations, finding identifiable themes and largely favorable sentiment toward ChatGPT (Xu et al., 2024). Cai et al. (2023) similarly analyzed Reddit’s mental health communities, using BERTopic to show how sentiment around ChatGPT shifted over time (Cai et al., 2023). Beyond social media discourse, systematic reviews and meta-analyses have investigated ChatGPT’s impact in domains such as education (Wang and Fan, 2025). These findings demonstrate that AI platforms are actively shaping both online communities and broader social practices. Several other large language models, including Anthropic’s Claude, xAI’s Grok, and Google’s Gemini have gained recent attention (Wangsa et al., 2024). Notably, Gemini has been integrated directly into Reddit through the Reddit Answers feature, (Huynh and Aichner, 2025; Klingbeil et al., 2024). A benchmarking study of eight chatbots, including Grok, Gemini, and DeepSeek, found that while exaggerated references were common, Grok and DeepSeek exhibited relatively fewer fabrications compared with others (Cabezas-Clavijo and Sidorenko-Bautista, 2025). Nonetheless, research on non-Western large language models remains scarce. DeepSeek’s Chinese roots offer a particularly insightful case for exploring transparency, trust, and openness within global AI ecosystems, where the concept of “open source” intersects with various regulatory and cultural expectations (Poo, 2025; White et al., 2024; Yang, 2025).

### 2.6 Research gap and contribution

While sentiment analysis, topic modeling, and network analysis have been widely applied to online communities, their integration, especially in subreddit-based studies of emerging AI models, remains underexplored. Previous research tends to isolate emotional, thematic, and interactional dimensions rather than examining their dynamic interaction within the same discursive space (Albalawi et al., 2020; Nandwani and Verma, 2021). Existing research largely focuses on Western-developed large language models like ChatGPT, overlooking non-Western initiatives that raise questions of transparency, governance, and trust. DeepSeek’s Chinese origin provides insight into the geopolitical dynamics of “open-source” AI amid differing cultural and regulatory norms (Poo, 2025; White et al., 2024).

Therefore, our research seeks to advance methodological integration within a novel sociotechnical framework by incorporating DeepSeek into global conversations. We offer one of the initial empirical mappings of how emotional, thematic, and relational cues intersect in the public’s perception of a Chinese open-source LLM, thereby contributing to discussions on AI governance, legitimacy, and trust.

This study contributes in three main aspects:

1. We analyze both posts and comments in r/deepseek, capturing not only topical structures but also the sentiments expressed by users and authors.
2. We construct a directed interaction network that segregates communities by topic clusters and overlays sentiment, mapping how emotions circulate.
3. We conduct a domain-level hyperlink analysis to reveal the external resources shaping AI-specific discourse.

Together, these contributions provide one of the first systematic analyses of community discourse on DeepSeek, offering a multidimensional view that integrates sentiment, thematic, and structural perspectives. This framework is reproducible and adaptable for studying other technology-focused subreddits.

## 3 Methodology

### 3.1 Data collection

The dataset was obtained from the r/deepseek subreddit, consisting of posts and comments from January 2025 to May 2025, utilizing the open-source Project Arctic Shift tool (Heitmann, 2024). The analysis spanned from January to May 2025, starting with the launch of DeepSeek R1 and continuing through its immediate aftermath to capture public attention and the subsequent post-release discourse. This tool facilitated filtering by subreddit and time period, with the data exported in JSONL format. For increased compatibility with Python and Microsoft Excel, JSONL files were converted to CSV. The data preparation steps, as outlined by Goel et al. (2023), are summarized in the Table 1 to provide a clear overview of the process.

**Table 1:** Overview of data pre-processing pipeline: this table details the preprocessing steps for r/deepseek posts and comments (January–May 2025).

Step	Description	Tools/techniques
Data acquisition	Collected posts and comments from r/deepseek (Jan-May 2025) in JSONL format.	Project Arctic Shift
Format conversion	Transformed JSONL files into CSV for easier analysis.	*json* and *pandas* Python libraries
Data purification	Removed deleted posts/comments and comments tied to deleted parent posts.	Python-based filtering
Text standardization	Converted text to lowercase, removed punctuation, URLs, emails, and hashtags.	Python text processing
Word normalization	Standardized elongated words (e.g., “goooood” to “good”) and expanded social media abbreviations.	Python text normalization
Stop word removal	Eliminated non-essential words (e.g., “and,” “the”) irrelevant to sentiment.	NLTK stopwords
Spelling correction	Detected and corrected spelling errors in the text.	Spellchecker library
Lemmatization	Reduced words to their root forms (e.g., “running” to “run”) for consistency.	NLTK WordNetLemmatizer

### 3.2 Sentiment and emotion analysis

Sentiment of the posts and comments were evaluated using the Valence Aware Dictionary and sEntiment Reasoner (VADER) from NLTK (Wankhade et al., 2022), a lexicon-based method. VADER is optimized for short, informal, and emotive online texts like Reddit posts, providing reliable sentiment estimates without fine-tuning large models (Hutto and Gilbert, 2014), ensuring transparency and reproducibility. Compared with transformer-based classifiers (e.g., BERT, RoBERTa), VADER requires minimal computation and no labeled data, making it well suited for large-scale, exploratory analyses of open online discourse and hence it was employed in our study.

VADER assigns compound scores from −1 to +1, classifying the sentiment of text as negative (−1 to −0.05), neutral (−0.05 to +0.05), or positive (+0.05 to +1) based on the score. For emotion analysis, we employed a pre-trained model by Hartmann (2022), which identifies Ekman’s six basic emotions (anger, disgust, fear, joy, sadness, surprise) and a neutral category. Each post or comment was labeled with the emotion exhibiting the highest probability score.

### 3.3 Theme and topic modeling

Word clouds were generated using the wordcloud Python library (Mueller, 2020) to visualize frequently occurring terms, providing an overview of the themes in the posts and comments.

To identify key discussion themes, we employed BERTopic (Grootendorst, 2022), a comprehensive topic modeling framework. BERTopic was chosen for its ability to combine transformer embeddings with clustering and class-based TF-IDF to produce interpretable topic representations. In contrast to fully black-box neural topic models, BERTopic’s hybrid structure aligns with our goal of conducting a transparent, reproducible, and computationally efficient analysis of Reddit discussions. In our initial analysis, the text was transformed into numerical embeddings utilizing Sentence Transformers (Reimers and Gurevych, 2019), followed by dimensionality reduction through Uniform Manifold Approximation and Projection (UMAP). The embeddings were subsequently clustered using Hierarchical Density-Based Spatial Clustering of Applications with Noise (HDBSCAN). A token count matrix, produced with Scikit-learn’s CountVectorizer (Pedregosa et al., 2011), was used as input for BERTopic to extract the top 10 topics.

### 3.4 Hyperlink-based analysis

Links embedded in posts and comments were extracted to assess the external resources influencing DeepSeek discussions. Utilizing Python’s “requests” library, we verified the links by confirming a 200 (OK) status code, retaining only successful requests for further analysis. Notably, hyperlink frequencies were not normalized, as the analysis aimed to capture overall link prominence and naturally occurring visibility patterns in Reddit discussions. Additionally, we examined link-sharing patterns to identify popular and reliable sources, such as frequently cited domains, thereby providing insights into the external information impacting the community.

### 3.5 Network analysis

User interactions were modeled as a network, with users as nodes and their interactions (via posts and comments) as directed edges weighted based on the interaction frequency. Each comment and post was analyzed for sentiment using VADER and for topics using BERTopic. Post topics were inferred from the dominant topic among their associated comments. A CSV file was generated, containing columns for author, parent element, and sentiment. This file was visualized using Cytoscape (Shannon, 2003) to create a directed network graph. Networks with five or fewer nodes were excluded, and nodes were color-coded based on sentiment to highlight interaction patterns.

## 4 Results

### 4.1 Overview of the dataset

**Figure 1:**
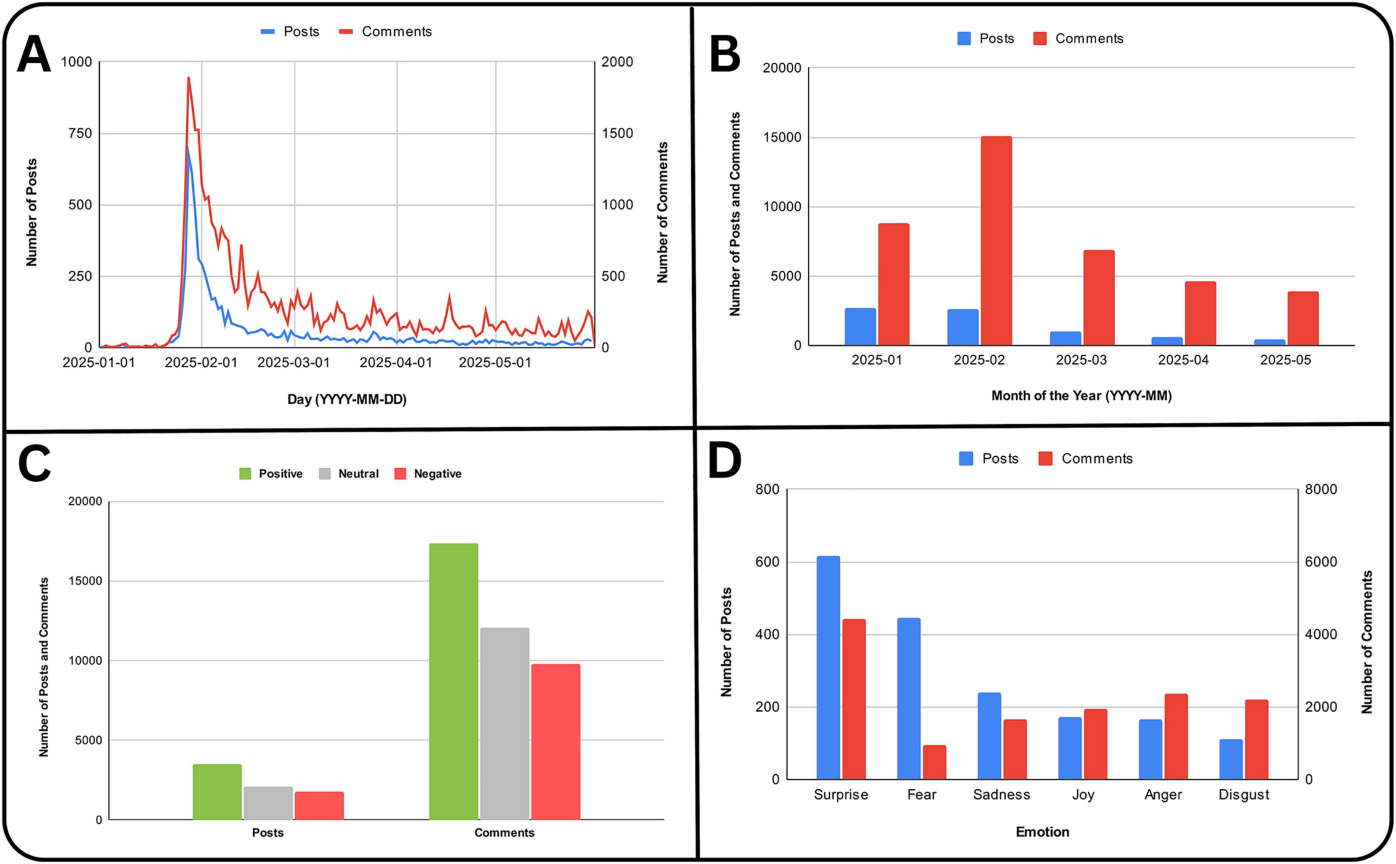
**(A)** Number of Posts and Comments in the dataset since the inception of the subreddit (r/deepseek). **(B)** Number of Posts and Comments in the dataset for the months January and May 2025. **(C)** Sentiment analysis of the Posts and Comments in the subreddit. **(D)** Emotion analysis of the Posts and Comments in the subreddit.

The data analyzed in this study were collected from Reddit’s r/deepseek subreddit, which was initially created on 29 November 2023 but remained largely inactive during its early months. Activity on the subreddit accelerated significantly following the public release of the DeepSeek-R1 model and its associated research paper on 22 January 2025 (DeepSeek-AI et al., 2025). This moment represented a significant shift, transforming r/deepseek into an active community platform where users engaged in spontaneous discussions, critiques, and experimental activities. The volume of posts and comments saw a substantial increase from January to February 2025 and declined after that until May 2025, reaching its peak shortly after the model’s introduction (Figures 1A,B). According to Google Trends data (Google Trends, 2015), public search interest in DeepSeek also rose sharply during this period, coinciding with heightened media coverage. This temporal overlap suggests that broader media attention and public curiosity may have contributed to the surge of Reddit activity surrounding the model’s release.

In total, the dataset included 7,400 posts and 39,249 comments made during this five-month surge. The subreddit operated as a hybrid space, simultaneously functioning as a help forum, technical feedback channel, and cultural space where users debated the implications of DeepSeek’s open-source architecture, performance benchmarks, and Chinese origin.

### 4.2 Sentiment and emotion analysis

Sentiment analysis was performed on posts and comments from the DeepSeek subreddit, collected between January 2025 and May 2025, using the VADER (Valence Aware Dictionary and sEntiment Reasoner) tool. Of the posts analyzed, 47.23% (*n* = 3,495) were classified as positive, 24.39% (*n* = 1805) as negative, and 28.38% (*n* = 2,100) as neutral. For comments, 44.26% (*n* = 17,373) exhibited positive sentiment, 24.97% (*n* = 9,800) negative, and 30.77% (*n* = 12,076) neutral. These distributions, summarized in Table 2 and Figure 1C, indicate a prevalence of positive sentiment across both content types, with comments showing slightly higher neutrality. Some examples of the positive, negative and neutral posts and comments can be found in Table 3.

**Table 2:** Dataset statistics after pre-processing.

Sentiment	Positive	Neutral	Negative	Total
Posts	3,495	2,100	1805	7,400
Comments	17,373	12,076	9,800	39,249
Total	20,868	14,176	11,605	46,649

**Table 3:** Examples of posts annotated as positive, negative, or neutral sentiments.

Sentiment	Total posts and comments	Examples
Positive	20,868 (43.73%)	AI models are getting better at logic, problem-solving, and even generating creative content. But true reasoning and creativity still feel like human-dominated areas. DeepSeek and similar models are making progress so how far can this go? Do you think AI will ever truly reason like humans, or will it always just mimic patterns? Where do you see the biggest challenges? Let us discuss! The thing is superb, the reasoning is like having 10gpts working together, i have seen benchmarks of even the best O3 and its not that far even though it’s very expensive. What makes it super is the reasoning design, the thing thinks lols and it does great at most problems. Today i was fixing a fridge and i used deepseek to think about my problems and the thing was on, it’s like having another mind next to me thinking.
Neutral	14,176 (30.39%)	What’s your setup and what are your speeds? It would provide a very detailed answer but when I go to read the response, it changes to the above. Hi folks, I just tried and documented the steps to install deepSeek locally, take a look at this blog.
Negative	11,605 (24.88%)	Stop talking about deepseek now. Every prompt is rejected with sorry DeepSeek is experiencing high traffic. You guys ruined it for us Guys i just wanted to tell for people who started using deepseek, dont use it its just another bad chatgpt copy,servers are always busy and his answers are SHIT! If the US federal government blocks DeepSeek on government phones than I’d suspect DeepSeek is exactly what people need. This is a government full of corrupt bastards so whatever they say, I’ll do the opposite.

Emotion analysis showed that neutral emotion was most prevalent, appearing in 60% of posts and 65% of comments. Surprise and fear were the leading non-neutral emotions (Figure 1D; Supplementary Figure 1). Anger and disgust appeared more often in comments, while fear was less frequent. This pattern suggests that most discussions were technical or informational, with emotional responses such as surprise and fear reflecting users’ reactions to DeepSeek’s capabilities and implications.

### 4.3 Theme and topic modeling

**Figure 2:**
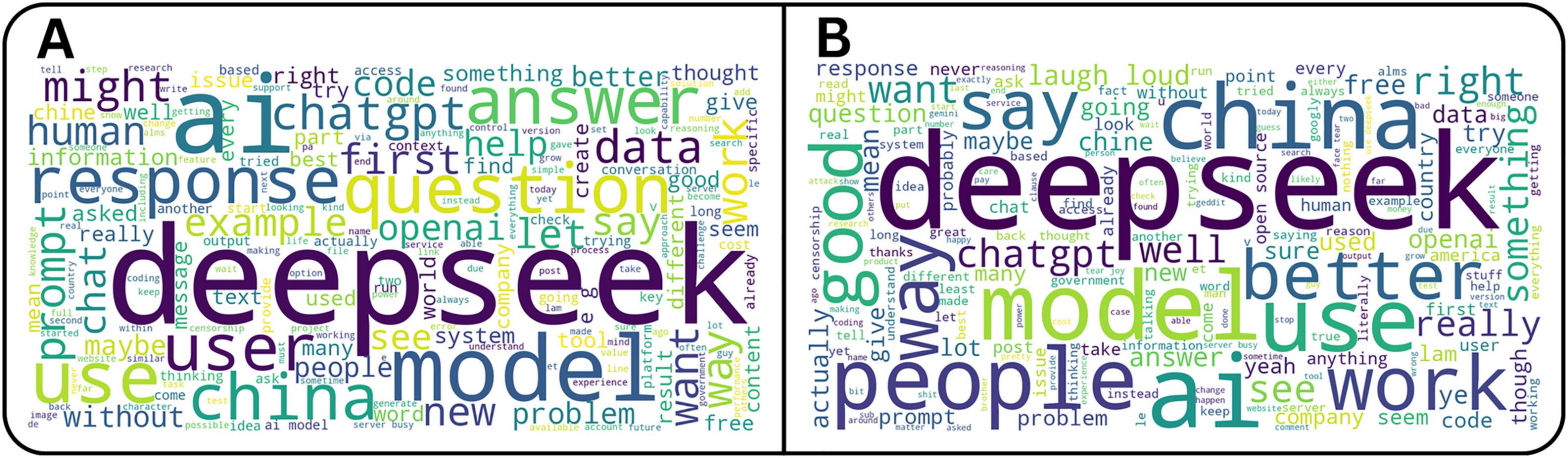
Word-cloud analysis of social media activity. **(A)** Word cloud of the most frequent terms extracted from posts. **(B)** Word cloud of the most frequent terms extracted from comments. Prominent recurring terms such as deepseek, model, use, and China reveal dominant themes and key areas of discussion across both posts and comment.

To visualize overall thematic patterns, word clouds were generated for both posts and comments (Figure 2). The most frequently occurring terms were “deepseek,” “model,” and “AI,” reflecting the subreddit’s primary focus. Other frequent words such as “china,” “question,” and “better” indicate that discussions extended beyond technical evaluation to include comparative and critical perspectives.

To investigate the discursive environment of the r/deepseek subreddit, we applied topic modeling techniques to identify common themes in user posts and comments. Using BERTopic, a framework for contextual language modeling, we extracted the top 10 discussion topics from the dataset (Table 4). This method allowed us to look beyond individual keywords and understand how users collectively perceived the technological, political, and infrastructural elements of DeepSeek.

**Table 4:** Top 10 topics extracted from the comments of r/deepseek subreddit.

Topic	Count	Title	Description
1	17,719	Open Source AI Models	This topic centers on the public availability of the DeepSeek model weights, allowing users to run the model on their systems.
2	511	DeepSeek Chat Usage	Users discussed the DeepSeek website and how anyone can interact with the DeepSeek model directly through the chat interface.
3	476	Email Accounts and Services	Focuses on the requirement of email registration to access the chatbot, leading to discussions around account creation and email services.
4	450	Android Phones and Mobile Web	Discussions around accessing AI models, especially DeepSeek Chat, using mobile devices such as Android phones and mobile browsers.
5	369	Internet Search Behavior	Covers how users find and access AI tools like DeepSeek Chat via search engines and general online discovery behavior.
6	315	ChatGPT and DeepSeek User Opinions	Users compare ChatGPT and DeepSeek, sharing opinions, preferences, and performance comparisons.
7	312	OpenAI Discussion and Feedback	Involves feedback, criticism, or praise directed at OpenAI, its tools, policies, and research direction.
8	305	Laughter and Humor Reactions	Comments expressing amusement, laughter, or humorous content encountered during interactions with the chatbot.
9	286	AI Capabilities and Use	General discussions on what AI models can do, including examples, potential applications, and observed limitations.
10	272	Online Censorship Concerns	Conversations about moderation, filtering, and perceived censorship of China government and Taiwan.

The most discussed topics can be found below:

**Open Source AI Models**: the most dominant topic centers on the extensive dialog regarding DeepSeek’s initiative to release its model weights and training code to the public, distinguishing it from more proprietary corporate AI platforms. Users expressed excitement about the accessibility and transparency offered by DeepSeek, while also acknowledging the practical challenges of running such large-scale models on local machines. Despite the project’s open-source status, commenters highlighted the significant storage and computational resources required, sparking discussions about who truly benefits from the open-source designation (Rahman et al., 2017).

**DeepSeek Chat Usage**: Another significant topic where users shared advice and appreciated the high usage limits for the model and about the model’s web and mobile interfaces. The requirement for email registration to use the chatbot led to a separate discussion cluster under the theme “Email Accounts and Services,” highlighting the tension between openness and platform control. Additional themes included device-specific usage (“Android Phones and Mobile Web”), search behavior, and comparisons with other apps like ChatGPT. These discussions often combined personal experimentation with broader critiques, making Reddit a space for active engagement with infrastructure.

**Online Censorship Concerns**: Topic modeling revealed politically charged discourse, users noted that DeepSeek systematically avoided or blocked responses to questions referencing the Chinese government or Taiwan, raising suspicions about training data restrictions and output-level censorship (Yang, 2025). Unlike Western AI models, which often restrict illegal or copyrighted content post-generation, DeepSeek’s filtering appeared embedded into both training and response layers, reinforcing anxieties about ideological influence.

**Laughter and Humor Reactions**: Humor and sarcasm are known to be prevalent on Reddit, where irony, memes, and playful exchanges are integral to the platform’s communication style (Biri and Tanskanen, 2025; Liebeskind and Bączkowska, 2025). While overt trolling is moderated, witty posts often gain high engagement. In r/deepseek, humor-related content indicates that playfulness helps users navigate technical complexity, fostering community identity, and commenting on AI hype. This trend mirrors Reddit’s broader norm, where irony and amusement coexist with serious discussion, shaping both the tone and participation patterns of discourse.

Overall, topic modeling reveals that r/deepseek was not merely for troubleshooting or praise, but a discursive arena where users evaluated DeepSeek as both tool and symbol, negotiating its implications within broader narratives about AI ethics, open-source access, and global power structures.

**Figure 3:**
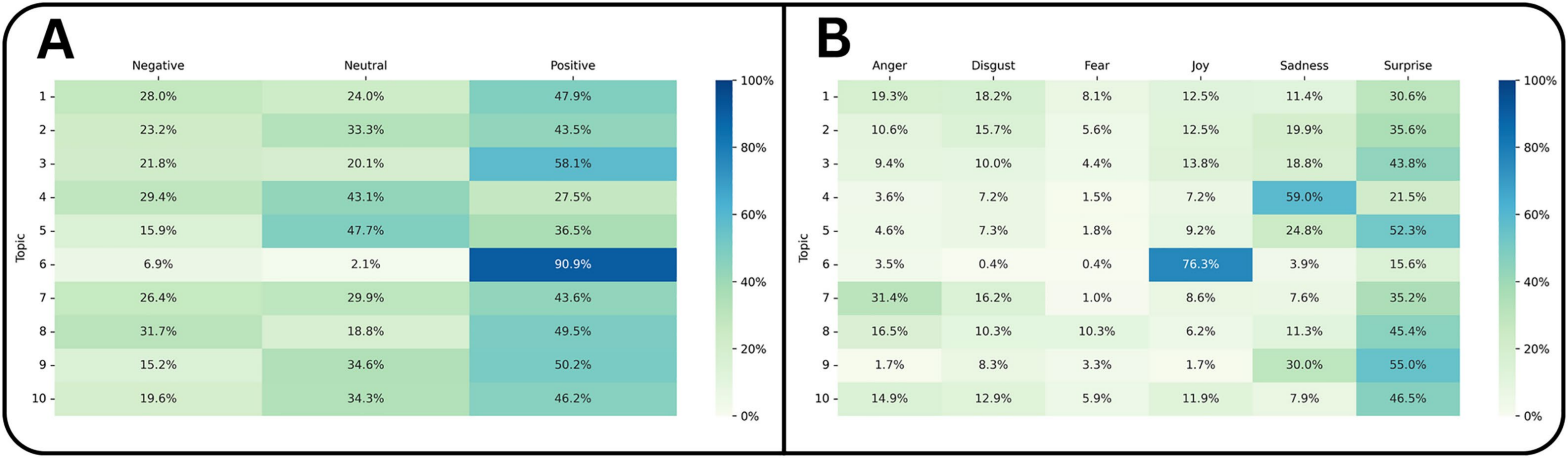
Heatmaps depicting the correlation of sentiment and emotion across topics in Reddit discussions about DeepSeek. **(A)** Proportion of negative, neutral, positive sentiment per topic. **(B)** Proportion of six basic emotions (anger, disgust, fear, joy, sadness, surprise) per topic. Darker shading indicates higher relative frequency within each sentiment or emotion category.

Figure 3 presents the heatmaps of sentiments and emotions across various topics. Overall, most topics predominantly exhibited positive sentiment, followed by neutral and negative tones. Topic 6 (“ChatGPT and DeepSeek User Opinions”) was overwhelmingly positive (≈90.9%), reflecting users’ praise for DeepSeek’s performance compared to ChatGPT. In contrast, Topic 8 (“Laughter and Humor Reactions”) included memes and ironic remarks, which VADER often classified as negative, likely due to sarcasm (Liebeskind and Bączkowska, 2025). Topics 4 and 5 (“Internet and Phones”) showed a higher proportion of neutral sentiment, aligning with more general, less opinionated discussions. For emotion analysis (Figure 3B), Topic 6 again demonstrated a dominance of joy, corresponding to enthusiasm for DeepSeek’s accessibility and capabilities. Sadness and surprise were the next most frequent emotions overall, often linked to conversations about AI limitations, censorship, or unexpected outputs. Topic 7 (“Feedback for OpenAI”) displayed elevated anger, reflecting user frustration over ChatGPT’s perceived drawbacks, while surprise was most pronounced in “AI Capabilities and Use,” indicating genuine amazement at DeepSeek’s technical potential.

### 4.4 Hyperlink-based analysis

This study extracted a total of 3,304 hyperlinks, with 1,134 extracted from posts and 2,170 from comments. Table 5 presents the top 10 hyperlinks along with a brief description. Most of the hyperlinks direct to https://preview.redd.it, which is where Reddit stores and retrieves user-uploaded images. Links to https://www.reddit.com are used for sharing and discussing other Reddit posts and comments, whether from the same subreddit or different ones. The URLs https://github.com and https://huggingface.co host the codes and the model weights. The link https://status.deepseek.com is used to verify if the site is operational or if the servers are overloaded and have crashed. On https://medium.com and https://x.com, users share guides, news, and opinions. The platform https://ollama.com facilitates running open-source models like deepseek on local systems. Lastly, https://chat.deepseek.com allows users to access deepseek for free, provided they register with a valid email address.

**Table 5:** Analysis of the hyperlinks extracted from the posts and comments of the r/deepseek subreddit.

Domain	Posts	Comments	Total	Website description
https://preview.redd.it	444	762	1,206	Reddit image preview link
https://www.reddit.com	53	209	262	Social news and discussion platform
https://www.youtube.com	78	106	184	Video sharing and streaming platform
https://github.com	75	79	154	Code hosting and collaboration for developers
https://huggingface.co	12	54	66	Platform for AI models and NLP tools
https://status.deepseek.com	9	50	59	DeepSeek service status dashboard
https://medium.com	7	42	49	Online publishing platform for articles
https://x.com	35	9	44	Social media platform (formerly Twitter)
https://ollama.com	9	35	44	Local LLM deployment and inference tool
https://chat.deepseek.com	22	21	43	DeepSeek’s AI chatbot interface

### 4.5 Network analysis

**Figure 4:**
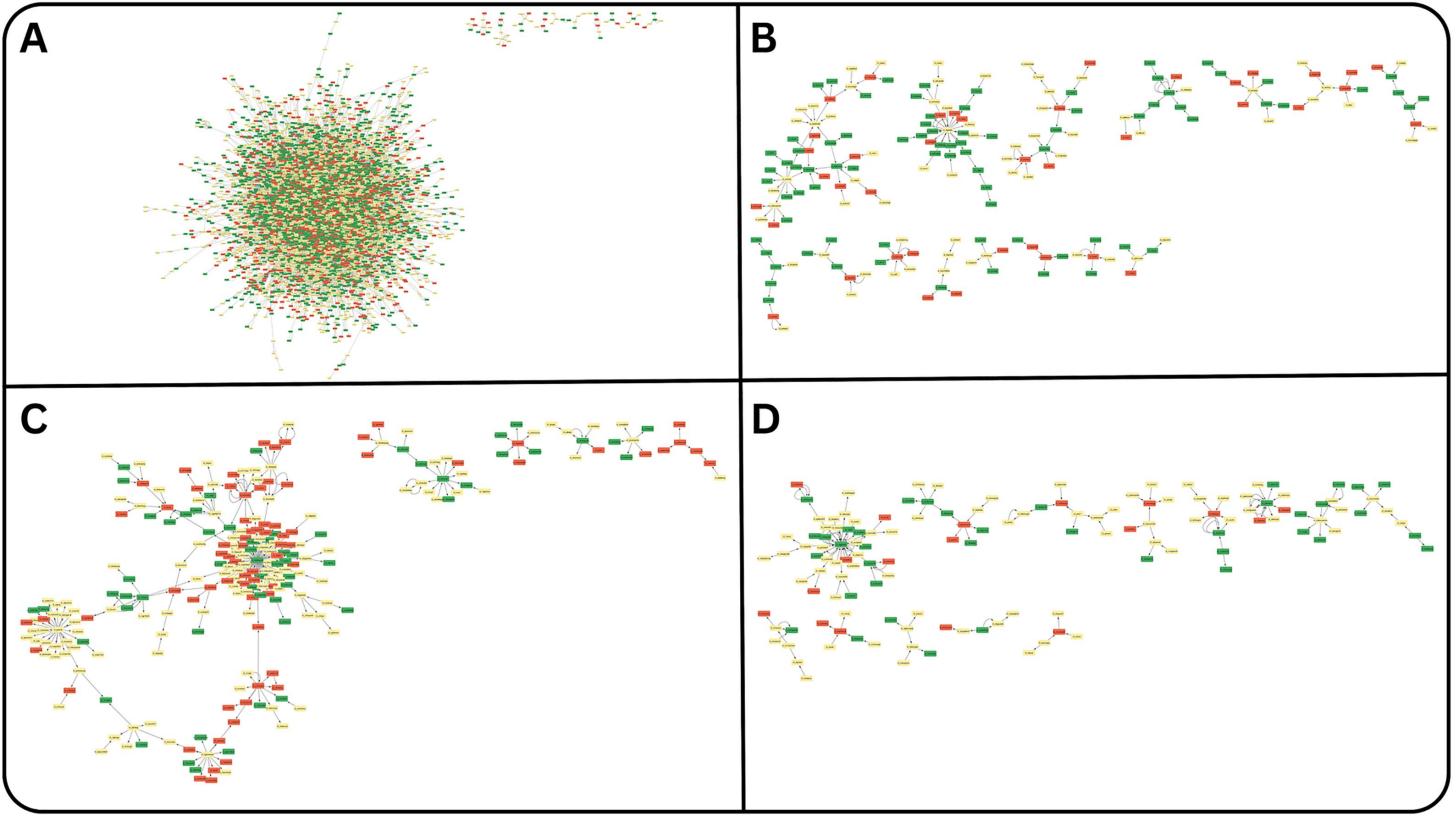
Network analysis of the authors of the posts and comments of the r/deepseek subreddit clustered based on the topics. **(A)** Network of the authors for Topic-1: Open Source AI models. **(B)** Network of the authors for Topic-2: DeepSeek chat usage. **(C)** Network of the authors for Topic-3: email accounts and services. **(D)** Network of the authors for Topic-4: android phones and mobile web.

To analyze interaction patterns within the r/deepseek subreddit, we created directed user-comment networks based on replies, where nodes symbolize users and edges represent posting and commenting activity. The larger networks (≥5 nodes) for each topic are shown in Figures 4A–D, while the complete networks are provided in Supplementary Figures 2–5. Sentiment labels were assigned to nodes according to the sentiment of their contributions, and visualizations were produced using Cytoscape. The largest and most densely connected network formed around Topic 1 – Open Source AI Models, highlighting both the popularity and technical complexity of discussions about DeepSeek’s architecture. Within this cluster, a few central users attracted numerous responses, acting as informal hubs of expertise or influence. These users often wrote explanatory posts or shared practical resources (e.g., GitHub links, installation guides), leading to cascades of comments, clarifications, and follow-up questions. Other topics, such as “Email Accounts and Services” or “DeepSeek Chat Usage,” generated smaller, closely-knit networks. These resembled temporary micro-communities, where a core group of users interacted repeatedly within a specific thread or theme. These interactional clusters demonstrate how Reddit facilitates episodic public users temporarily gathering around a technical concern, then dispersing. Many networks exhibited shallow structures, with just two to four nodes, typically a user posting a question and receiving one or two responses. These minimalist threads suggest Reddit’s role as both a broadcast medium and asynchronous help desk, particularly for users engaging with DeepSeek for the first time. Interestingly, most topic networks showed low cross-topic connectivity, that is, users discussing censorship did not typically engage in technical debates, and vice versa. This fragmentation may indicate parallel publics forming within the same subreddit, each driven by distinct interests (e.g., infrastructure, politics, user experience). Sentiment overlay further revealed that these micro-networks did not form clear ideological or emotional echo chambers. Similar to patterns reported in political subreddits (De Francisci Morales et al., 2021), r/deepseek networks displayed sentiment heterogeneity: positive and negative responses often coexisted within the same thread. This suggests that users were not merely reinforcing shared affective positions, but engaging in negotiation, challenging claims, clarifying expectations, and offering support within a loosely threaded discourse structure. Taken together, the network analysis portrays Reddit as a relationally diverse, sentimentally mixed, and topically segmented platform, where AI-related discourse unfolds through uneven patterns of interaction and temporary user clustering. These dynamics illustrate how users perform not just technical evaluation, but communal boundary-work, as they position themselves in relation to the DeepSeek model, to each other, and to broader debates about AI openness, control, and credibility.

## 5 Discussion

Our study aimed to examine how online communities interpret and assess emerging AI models, with a particular focus on DeepSeek, through discussions on Reddit. Recent advancements in generative AI, such as ChatGPT, Gemini, Claude, and LLaMA, have intensified debates surrounding automation, creativity, labor, and governance. Developed by major technology companies and trained on extensive public and private datasets, these systems continue to raise concerns about transparency, control, and accessibility. In the midst of these discussions, DeepSeek has emerged as a unique case: a Chinese-developed open-weights model released by the High-Flyer hedge fund, which claims competitive performance with modest computational resources. According to the Open Source Initiative (OSI), DeepSeek qualifies as an open-weights model because its codes and weights are public, although its training data remain proprietary (White et al., 2024; Hugging Face, 2025).

Sociotechnical imaginaries, collectively held visions of desirable futures, may influence how societies design and govern technologies (Jasanoff, 2015). Moreover, generative AI models like ChatGPT and DeepSeek reflect and reproduce distinct imaginaries of automation, creativity, and control, shaped by their political and institutional contexts. From a platform studies perspective, Reddit serves as a site of algorithmic sense making (Gillespie, 2018), where users interpret and contest these imaginaries through posts, humor, and critique. DeepSeek therefore occupies a place within a global landscape of algorithmic power, where openness, censorship, and legitimacy are negotiated through cultural imaginaries and participatory online evaluation. Its unexpected arrival has generated significant public interest on digital platforms, especially Reddit. Our examination of r/deepseek illustrates how Reddit functions as a platform for sociotechnical negotiation, where users not only assess DeepSeek’s technical capabilities but also consider its cultural, geopolitical, and infrastructural ramifications. Conversations were influenced not only by enthusiasm for DeepSeek’s performance but also by apprehensions and uncertainties about its origins, data privacy, and political affiliations. These emotional responses, though mainly neutral due to a discussion related to technology, others ranging from surprise and admiration to suspicion and fear, emerged in comments and were closely linked to users’ views of DeepSeek as a product of Chinese technological advancement.

The community’s approach to trust, efficiency, and control reveals a certain interpretive ambiguity. On one hand, numerous users praised DeepSeek for its substantial token limits and its potential to compete with OpenAI’s paid products (Koubâa et al., 2023). Conversely, issues arose concerning censorship and data sovereignty. The model’s reluctance to address topics related to the Chinese government or Taiwan, as extensively discussed on the subreddit, sparked concerns about anticipatory political filtering (Yang, 2025). Unlike Western models that apply moderation at the output level, DeepSeek seems to integrate these restrictions during both the training and inference phases, highlighting the intertwining of geopolitical ideology with technical design (Webster, 2025; Yang, 2025). Cross-cultural trust dynamics significantly influence how users assess emerging AI systems. Perceptions of Chinese-developed models, such as DeepSeek, intertwine with narratives surrounding state influence, data governance, and technological sovereignty, leading to skepticism about their transparency compared to Western counterparts (Cheng and Zeng, 2022; Roberts et al., 2020). In contrast, Western AI systems are often linked to corporate dominance and ethical controversies regarding the data used for model training, rather than government control (Crawford, 2021). These differences may suggest algorithmic legitimacy is contingent upon geopolitical context, regulatory culture, and the structures of public trust.

User discussions on the platform show how public discourse around AI models mirrors broader economic and technological narratives. The excitement about DeepSeek, amplified by media reports on its GPU efficiency, coincided with an 18% decline in NVIDIA’s stock value by January 27, 2025 (Reid and Harring, 2025; Nvidia, 2025). While this overlap does not imply causation but shows how online discussions, news cycles, and investor sentiment evolve in parallel within a shared information ecosystem (Desiderio et al., 2025). Additionally, Reddit functioned as more than just a space for assessment. It acted as a transmedia infrastructure: users often shared links to GitHub,^1^ Hugging Face,^2^ Medium, and X, creating a distributed knowledge network around DeepSeek. As highlighted in earlier research, such hyperlinking practices demonstrate how platforms like Reddit bridge formal and informal knowledge, allowing vernacular technocultures to thrive (Massanari, 2017; Esteve et al., 2020). Even though DeepSeek is open-source, users quickly pointed out the challenges of deploying the model locally, particularly the significant storage and RAM needs (Rahman et al., 2017). This ongoing debate between openness and accessibility was a recurring theme in discussions and may indicate a growing awareness that open source does not ensure democratization when infrastructural challenges remain significant (Liesenfeld and Dingemanse, 2024).

Vernacular governance examines how communities such as r/deepseek negotiate and manage emerging technologies outside formal institutional frameworks (Erdocia et al., 2025; Janssen, 2025). It highlights how everyday critique, comparison, and commentary function as forms of participatory oversight in digital environments. On r/deepseek, users enacted this governance by evaluating transparency, questioning censorship, and comparing DeepSeek with models like ChatGPT and Gemini. The subreddit thus operated as a venue where legitimacy and trust in AI were constructed through user-driven dialog rather than top-down regulation.

Finally, the network analysis underscored that while user engagement often clustered around technical or affective themes, these communities remained relationally diverse and sentimentally mixed. There were no clear ideological echo chambers as seen in political subreddits (De Francisci Morales et al., 2021). Instead, users interacted across sentiment lines, suggesting that AI discussions, especially around emergent models, retain an open-ended, deliberative character. Notably, many users interacted with only one other user, reflecting the prevalence of one-to-one exchanges on Reddit as previously documented (Goglia and Vega, 2024).

Taken together, this study positions Reddit not just as a site of user opinion, but as a media infrastructure for algorithmic reception and critique. DeepSeek’s reception reveals the layered ways in which users make sense of AI: as software, as political artifacts, and as symbols of shifting technological power. As generative AI continues to proliferate, such user-led discourses offer critical insight into how the public negotiates questions of trust, access, and agency within increasingly opaque AI ecosystems.

Although this study sheds light on the public discussion surrounding DeepSeek on Reddit, it is important to recognize several limitations. Firstly, our analysis is limited to a single subreddit (r/deepseek), which, despite its high activity during the launch period, only captures a portion of the broader conversation happening on platforms like X, Facebook and YouTube. Secondly, while sentiment and emotion classification are supported by established NLP tools, they may have difficulty identifying sarcasm, irony, or the platform-specific language often found on Reddit, such as meme-speak or downvoted critiques. Thirdly, although topic modeling is effective in identifying recurring themes, it does not account for the narrative flow or the argumentative structure of threaded discussions. Lastly, our data is restricted to the immediate post-launch period (Jan–May 2025), leaving long-term changes in perception and platform adaptation unexplored. Future research could employ longitudinal, cross-platform, or qualitative ethnographic methods to broaden and deepen our understanding of how AI technologies are perceived and reinterpreted by users in real time.

## 6 Conclusion

This study examined Reddit’s r/deepseek subreddit as a discourse site for the DeepSeek generative AI model. Through sentiment analysis, topic modeling, and network analysis, we revealed how users discussed the model’s performance and accessibility. By integrating affective, thematic, and relational perspectives, the study demonstrates how online communities collectively shape the legitimacy and interpretation of emerging AI models. We highlight a form of vernacular governance, where trust in AI is negotiated through everyday critique and peer evaluation on open platforms. These user-led interactions serve as informal regulatory practices that influence public understanding of transparency and credibility. Future research should enhance this framework by conducting longitudinal and multimodal analyses. This includes tracking sentiment dynamics over time, examining visual and multimodal Reddit content such as memes, screenshots, and images, and comparing discourse across platforms like X or Discord to capture broader sociotechnical trends. Overall, our findings show that public discourse plays an active role in shaping trust, accountability, and legitimacy within responsible AI governance.

## Data Availability

Publicly available datasets were analyzed in this study. This data can be found at: https://www.reddit.com/ and downloaded using https://github.com/ArthurHeitmann/arctic_shift.

## Footnotes

1
https://github.com/deepseek-ai

2
https://huggingface.co/chat
