# Supplementary material for: Understanding user perceptions of DeepSeek: insights from sentiment, topic and network analysis using a Reddit-based study
Source: Front Artif Intell. 2026 Jan 6;8:1703949. doi: 10.3389/frai.2025.1703949 (PMC12816320; doi:10.3389/frai.2025.1703949)
Supplement: Supplementary file 1 [file Data_Sheet_1.docx]

**Supplementary File – 1**

**Understanding User Perceptions of DeepSeek: Insights from Sentiment, Topic and Network Analysis Using a Reddit-Based Study**

Naisarg Patel^1^, Rajesh Sharma^2,3^, Prakash Lingasamy^4,5^, Vino Sundararajan^1^, Sajitha Lulu. S*^1^, Vijayachitra Modhukur^4^

1. Integrative Multiomics Lab, School of Bio Sciences and Technology, Vellore Institute of Technology, Vellore – 632014, Tamil Nadu, India
2. School of AI and Computer Science, Plaksha University, Punjab, India
3. Institute of Computer Science, University of Tartu, 51009 Tartu, Estonia
4. Department of Obstetrics and Gynecology, Institute of Clinical Medicine, University of Tartu, L. Puusepa 8, 50406 Tartu, Estonia
5. Nalam Biosciences OÜ, Tartu, Estonia

*Corresponding author:

Email: [ssajithalulu@vit.ac.in](mailto:ssajithalulu@vit.ac.in)

**Index:**

**Supplementary Figure. 1:** Emotion analysis of the Posts and Comments in the subreddit including the Neutral Class

**Supplementary Figure. 2:** Network visualization of user interactions in the Topic-1 from r/deepseek subreddit where nodes represent individual users, and edges represent comments interactions between them.

**Supplementary Figure. 3:** Network visualization of user interactions in the Topic-2 from r/deepseek subreddit where nodes represent individual users, and edges represent comments interactions between them.

**Supplementary Figure. 4:** Network visualization of user interactions in the Topic-3 from r/deepseek subreddit where nodes represent individual users, and edges represent comments interactions between them.

**Supplementary Figure. 5:** Network visualization of user interactions in the Topic-4 from r/deepseek subreddit where nodes represent individual users, and edges represent comments interactions between them.


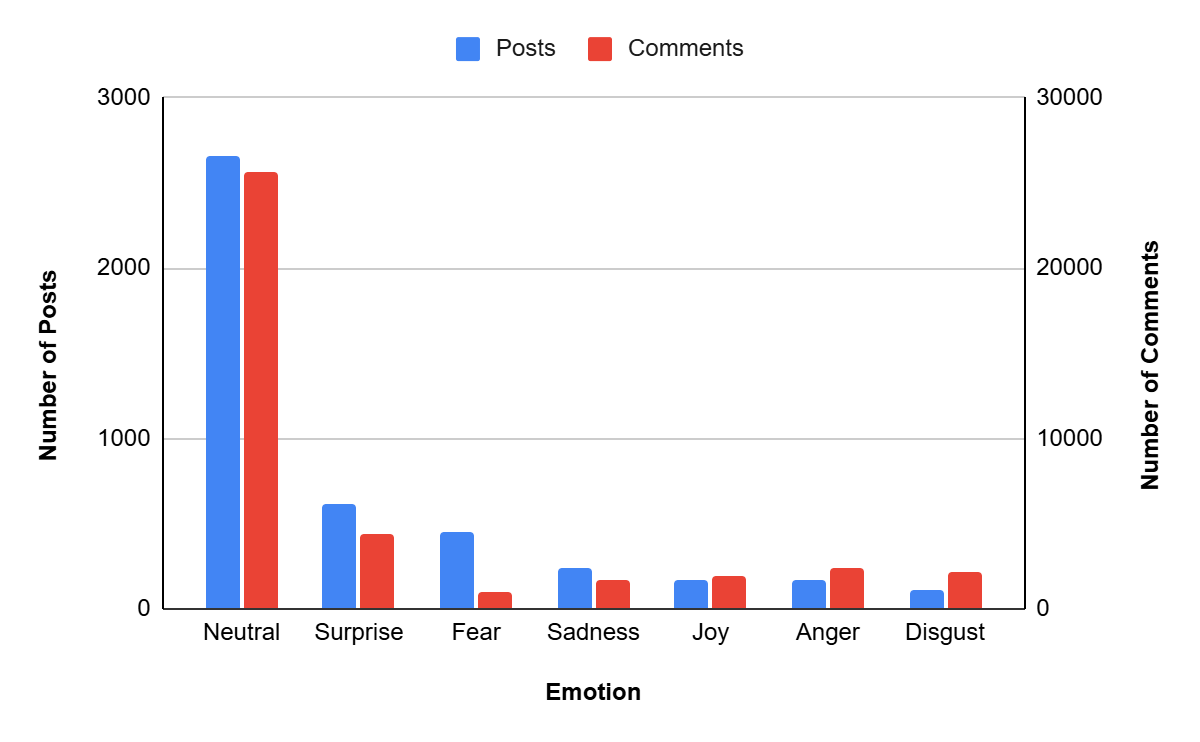


**Supplementary Figure. 1:** Emotion analysis of the Posts and Comments in the subreddit including the Neutral Class


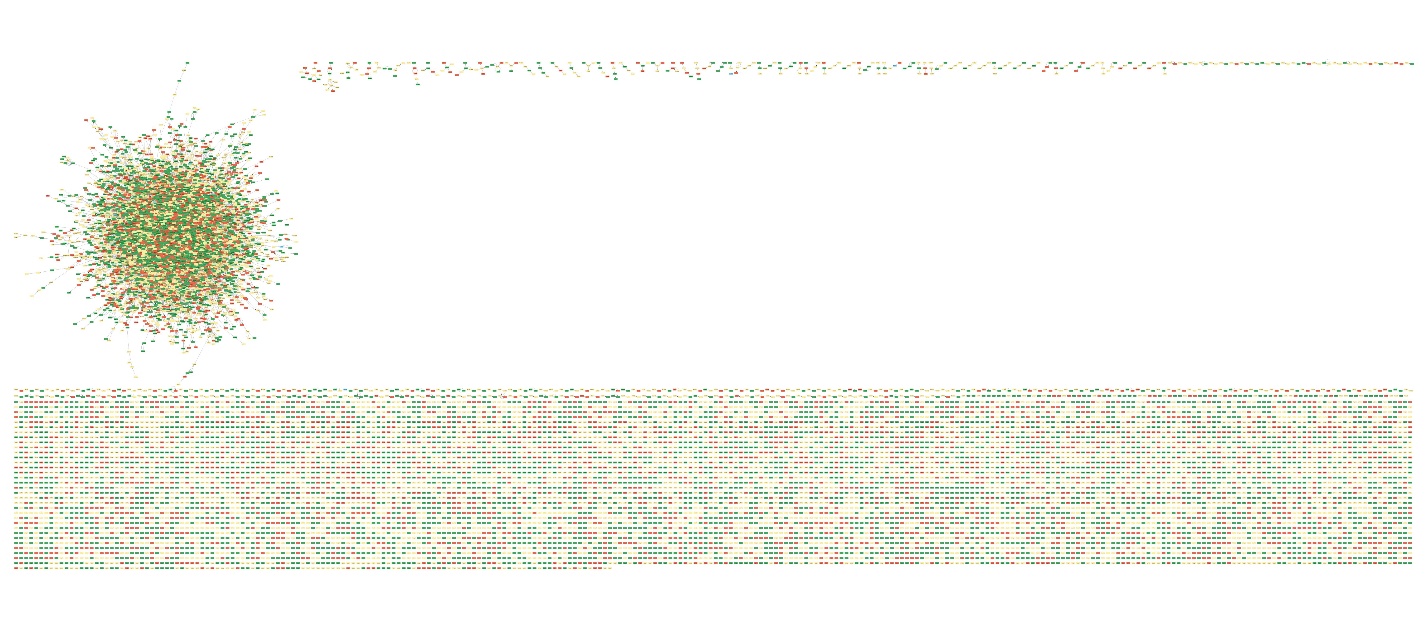


**Supplementary Figure. 2:** Network visualization of user interactions in the Topic-1 from r/deepseek subreddit where nodes represent individual users, and edges represent comments interactions between them.


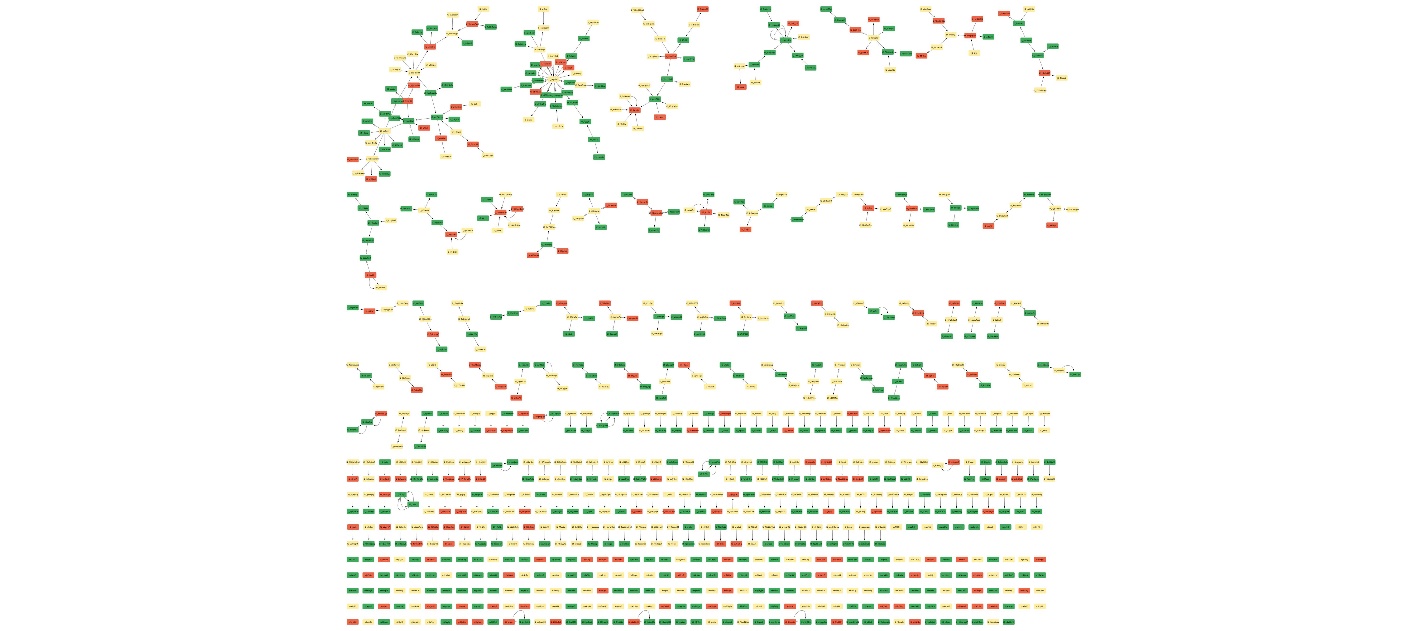


**Supplementary Figure. 3:** Network visualization of user interactions in the Topic-2 from r/deepseek subreddit where nodes represent individual users, and edges represent comments interactions between them.


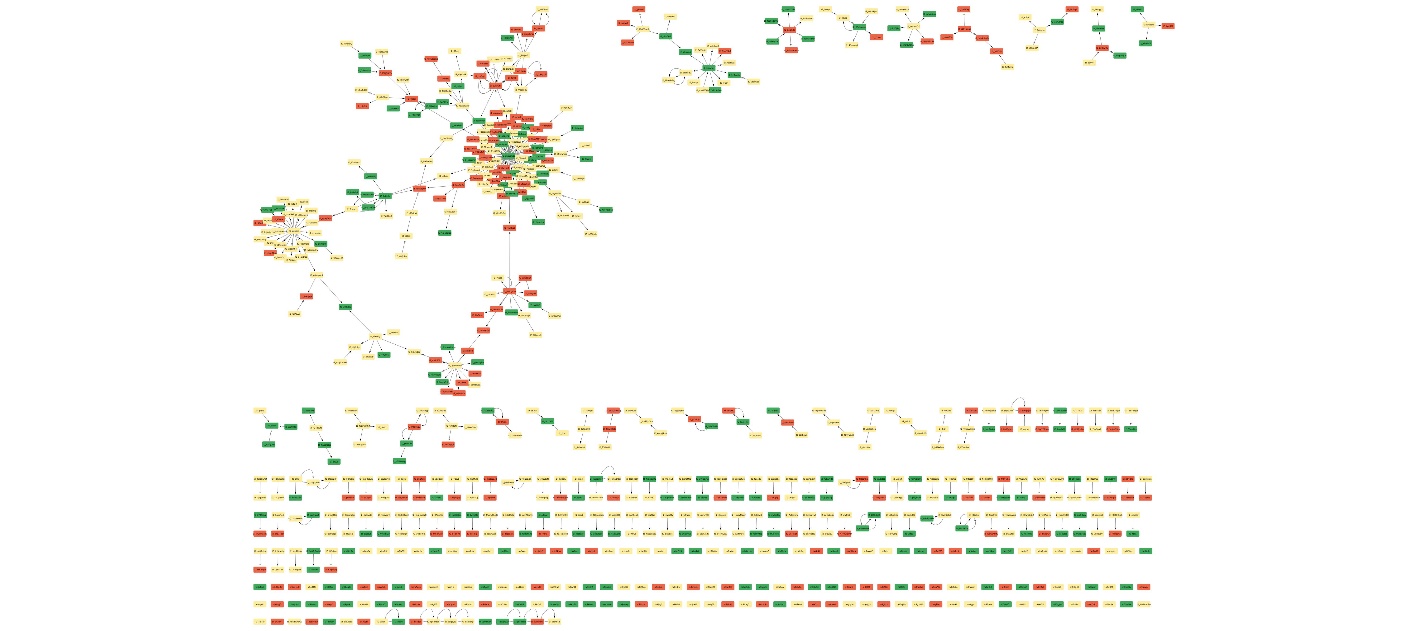


**Supplementary Figure. 4:** Network visualization of user interactions in the Topic-3 from r/deepseek subreddit where nodes represent individual users, and edges represent comments interactions between them.


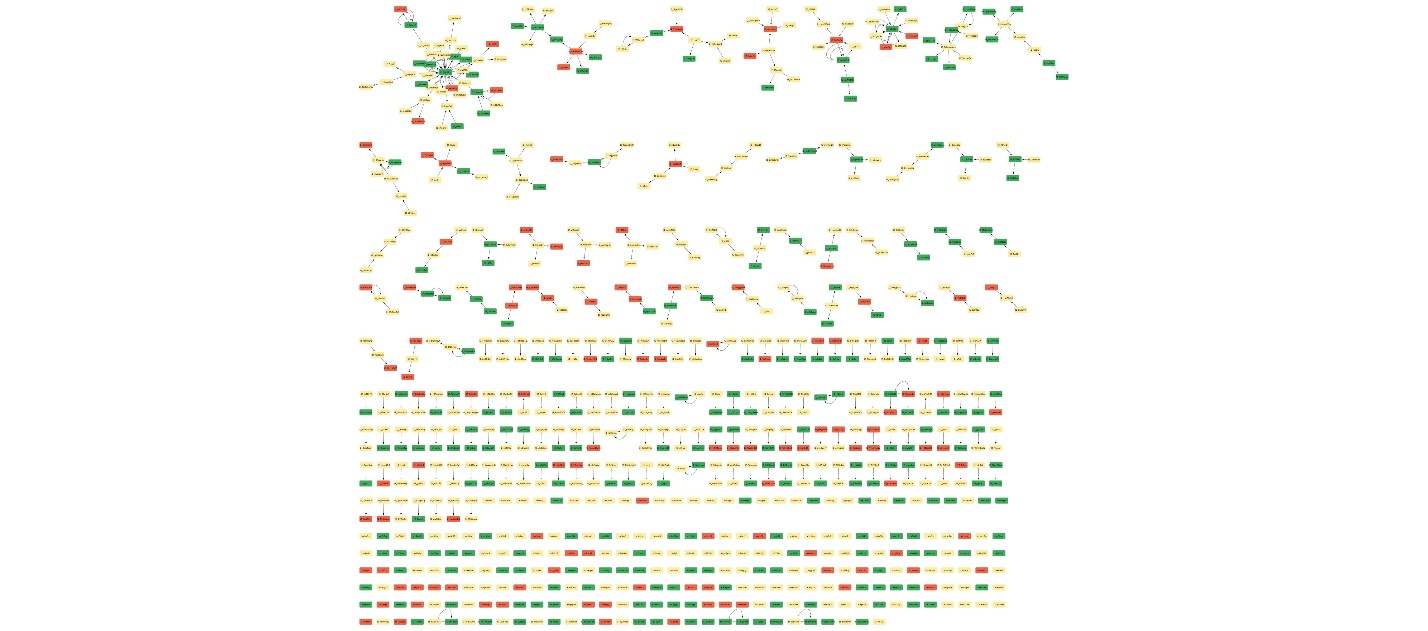


**Supplementary Figure. 5:** Network visualization of user interactions in the Topic-4 from r/deepseek subreddit where nodes represent individual users, and edges represent comments interactions between them.
